# An Investigation of MMR-Related Mumps Cluster Following Immunization Among Practical Nursing Students, Bangkok, Thailand, 2024

**DOI:** 10.1155/jotm/9974081

**Published:** 2025-08-01

**Authors:** Sethapong Lertsakulbunlue, Drunphob Srithammavong, Kamonchanok Tepsittha, Hataya Kanjanasombut, Vitchakorn Poonyakanok, Viravarn Luvira, Phimphan Pisutsan, Rachata Charoenwisedsil, Pathomthep Leowattana, Peeriya Watakulsin, Rapeepong Suphanchaimat, Atchariya Lukebua, Worawat Dangsagul, Kannikar Kwanchum, Thanit Rattanathumsakul, Pawinee Doungngern

**Affiliations:** ^1^Division of Epidemiology, Department of Disease Control, Ministry of Public Health, Nonthaburi 11000, Thailand; ^2^Department of Pharmacology, Phramongkutklao College of Medicine, Bangkok 10400, Thailand; ^3^Institute of Preventive Medicine, Department of Disease Control, Ministry of Public Health, Nonthaburi 11000, Thailand; ^4^Department of Clinical Tropical Medicine, Faculty of Tropical Medicine, Mahidol University, Bangkok 10400, Thailand; ^5^Office of Disease Prevention and Control 2, Ministry of Public Health, Phitsanulok 65000, Thailand; ^6^International Health Policy Foundation, Nonthaburi 11000, Thailand; ^7^National Institute of Health, Department of Medical Sciences, Ministry of Public Health, Nonthaburi 11000, Thailand

**Keywords:** adverse events, L-Zagreb, MMR vaccine, mumps, Thailand

## Abstract

**Background:** Although MMR vaccination can induce mumps infections, clustered cases right after the vaccination are rarely reported. On September 10, 2024, the Department of Disease Control, Thailand, received a report of a cluster of practical nursing students (PNSs) with jaw and ear swelling following an MMR (L-Zagreb strain) vaccination. An investigation was conducted to confirm the outbreak, identify sources and risk factors, and recommend preventive measures.

**Methods:** Active case findings were conducted among PNS and hospital service recipients who received the suspected vaccine batches. Suspected cases of MMR-related mumps infection were defined as individuals experiencing either jaw swelling/pain, testicular swelling/pain, or groin pain 12–25 days postvaccination. Confirmed cases had positive RT-PCR for mumps. *SH* gene sequencing determined mumps phylogenetics, while nanopore sequencing of the *NP* gene assessed polymorphisms. Group and in-depth interviews with vaccine suppliers, pharmacists, nurses, and PNS evaluated the vaccine cold chain and setting. A retrospective cohort study among PNS used questionnaires on demographics and vaccination history to identify risk factors, analyzed via multivariable logistic regression. Qualitative data underwent content analysis.

**Results:** Two batches of MMR vaccine were suspected and immediately suspended. Of the 108 vaccinated PNS, 12 met the case definition (eight suspected and four confirmed), yielding an attack rate of 11.1% (12.5% in males and 10.9% in females). Among 61 hospital vaccine recipients who received the same vaccine batches, 30 were contactable, and none met the case definition. All cases had ear/jaw pain, with 41.7% experiencing sore throats and 33.3% myalgia, though none were severe. Three confirmed cases had genetic material aligning with the L-Zagreb strain (Accession AY685920). No *NP* gene polymorphisms were detected in vaccines, though specimen buccal swabs had insufficient genetic material. Two factors may link to the adverse event: prior MMR vaccination (aOR = 12.90, 95% CI: 1.39, 172.00) and a delay of over 15 min from vial retrieval to administration (aOR = 26.90, 95% CI: 4.20, 247.00). Vaccine supply, distribution, and storage met standards, but vaccine campaign registration and waiting time processes require improvement.

**Conclusion:** MMR-related mumps infections were confirmed during PNS mass vaccination campaign. Potential risk factors include a history of prior MMR vaccination and delay time from vial retrieval to vaccine administration. Improvements are needed in vaccination campaigns, particularly in the registration system and expediting vaccination process flow.

## 1. Introduction

The Mumps–Measles–Rubella (MMR) vaccine has been used globally as a mandatory vaccine since the 1970s and was introduced in Thailand in 1984 [1, 2]. It contains live attenuated viruses, with the specific mumps strain varying by the country of manufacture, such as the Jeryl Lynn strain in the United States, the Urabe strain in Japan, the Leningrad-3 strain in Russia, and Rubini strain in Switzerland [3, 4]. Some of these strains have been associated with lower immunogenicity [5, 6], and the Rubini strain, in particular, has been documented as highly ineffective and is no longer recommended for use [4]. In contrast, the Leningrad-Zagreb (L-Zagreb) attenuated mumps strain, developed in Croatia in 1972 from the Leningrad-3 strain, is recognized for its high vaccine efficacy of 95%–100% [5, 6]. The Serum Institute of India has used the L-Zagreb strain to produce mumps vaccines since the 1980s.

In Thailand, the combined MMR vaccine, administered as a second dose, was introduced in the Expanded Program for Immunization (EPI) 1997, replacing single-component measles vaccines. The mumps strains available in vaccines include Jeryl Lynn and L-Zagreb. Since mumps, measles, and rubella can be contagious during the asymptomatic period, MMR vaccination is recommended for healthcare workers who are usually at high risk of exposure to reduce pathogen transmission [1, 7].

The adverse events following MMR vaccination could include fever, parotitis, and lymphadenopathy, affecting approximately 0.05%–0.70% of children under 6 years [8, 9]. However, several studies have reported a relatively higher incidence of adverse events, suspected to be iatrogenic mumps infections from the L-Zagreb strain, in adults and children over 6 years [3, 8, 10, 11]. The reported attack rates range from 0.5% to 15.1%, with rates exceeding 10% observed during mass vaccination campaigns.

MMR-related mumps infections in adults typically manifest within 12–25 days following MMR vaccination [3, 12]. The symptoms resemble wild-type mumps, including fever, swelling or pain in the jaw or cheek, and testicular swelling or pain. To date, no severe complications, such as intracerebral infections, pneumonia, or hemorrhagic conditions, have been reported [3, 8, 11]. Horizontal transmission among adults was documented in only one case, involving contact with one of nine patients who exhibited mumps-like symptoms among 1875 young adults vaccinated with the L-Zagreb MMR vaccine. However, this transmission was not confirmed by molecular methods [8].

The most recent outbreak of MMR-related mumps among young adults in Thailand was reported in 2010, in which the L-Zagreb strain was used. Despite this outbreak, which led to the L-Zagreb strain being labeled as not recommended for adults, it continues to be used in several hospitals to immunize healthcare workers due to its high vaccine efficacy at a lower cost [3, 5]. To our knowledge, MMR-related mumps infection is rare [3]. On 10 September 2024, the Department of Disease Control, Thailand, received a report of a cluster of practical nursing students (PNSs) presenting with swollen jaws, ear pain, and, in some cases, low-grade fever. These symptoms raised suspicions of adverse events following immunization (AEFI) related to an MMR vaccination campaign conducted before their enrollment in clinical training. Consequently, an outbreak investigation was initiated to (1) confirm the outbreak and diagnosis, (2) identify potential sources, (3) describe the epidemiological characteristics, (4) analyze risk factors, and (5) provide recommendations.

## 2. Methods

### 2.1. Settings

This event occurred among 108 PNSs, who participated in a vaccination campaign, at the Practical Nursing School, in one of the university hospitals. The vaccination plan included five vaccines administered in the following order: influenza vaccine, MMR vaccine, varicella vaccine (for those with negative immunity), hepatitis B vaccine (for those with negative immunity), and Tdap vaccine. The influenza vaccine had been administered approximately 1 month prior to this occasion. Two doses of the MMR vaccine were planned, with this being the first dose. The same protocol and vaccine regimen had been consistently implemented over the past 3 years. The vaccination campaign was conducted at a 250-bed tertiary care hospital, which was also responsible for vaccine supply management. These vaccines were routinely administered to both adult and pediatric patients at the hospital's outpatient department.

### 2.2. Epidemiologic Investigation

A total of 108 PNS received two batches of the MMR vaccine on August 21, 2024. An MMR-related adverse event suspected case was defined as an individual who received the alleged vaccine batches and experienced any of the following symptoms: fever, jaw swelling or pain, testicular swelling or pain, or groin pain 12–25 days postvaccination (between September 2 and 16, 2024, for PNS), based on the mumps incubation period. Confirmed cases included suspected cases who tested positive for mumps by reverse transcriptase polymerase chain reaction (RT-PCR).

Active case findings were conducted among PNS and hospital service recipients who received the suspected MMR vaccine batches at the hospital. For the PNS, an online self-administered questionnaire was distributed via social networks. The questionnaire was adapted from the AEFI investigation form in the Thai Surveillance and Response Guideline for AEFI, incorporating questions specific to the current setting [13]. It was iteratively validated by all authors before use. The questionnaire consisted of four sections: demographics, preparation before vaccination, vaccination setting and services, and previous immunization history. Phone calls were made to hospital service recipients who received the suspected batch of the MMR vaccine to inquire about any adverse events following vaccination.

Furthermore, to identify possible wild-type mumps outbreaks within the hospital, a review of patients' medical charts of those visiting the hospitals with mumps or mumps-like symptoms from July 1 to August 31, 2024, was conducted using the International Classification of Diseases, Tenth Revision (ICD-10). The review included the following codes: mumps (B26), orchitis/epididymitis (N45), diseases of salivary glands (K11), Acute sialadenitis (K112), jaw pain (R6884), and salpingitis/oophoritis (N70).

Subsequently, a retrospective cohort study was conducted to describe the epidemiological characteristics and identify possible risk factors associated with the outbreak among PNS. All 108 PNS students vaccinated on August 21, 2024, were included in the study. The practical nursing instructors distributed questionnaires via Google Forms. Based on a previous study of vaccine-related mumps infections in Thailand, with an attack rate of 9.3%, the sample size estimation required 61 participants to achieve an adequate sample size for proportion with 80% power with a 95% confidence interval (CI) [3]. Assuming a 10% nonresponse rate, at least 68 participants should be included.

### 2.3. Laboratory Study

To confirm the diagnosis, buccal swabs were collected from all suspected cases and tested for mumps using RT-PCR at the Department of Medical Sciences, Ministry of Public Health. Small hydrophobic protein (*SH*) gene sequencing was also performed to identify the mumps genotype and differentiate between wild-type and vaccine-related infections. Whole blood samples were also collected to test for mumps IgM and IgG antibodies. Although the suspected vaccine batches were collected, they were not retested for vaccine quality as they had already passed and met approved standard testing. The review based on previous testing conducted by the Department of Medical Sciences, Ministry of Public Health, confirmed that the sterility, toxicity, and viral contents were up to standard.

#### 2.3.1. Amplicon-Based Nanopore Sequencing of Mumps NP Gene

The patient's buccal swabs and collected vaccine samples were further tested for a plausible *NP* gene polymorphism, a polymorphism previously reported in an MMR-related mumps infection [3]. Buccal swabs and isolated viruses were extracted using a QIAamp viral RNA mini kit (Qiagen, Hilden, Germany) per manufacturer's instructions. The RNA was eluted in 60-μL elution buffer and stored at −70°C until use. A set of primers to amplify the *NP* gene of mumps was designed by PrimalScheme v3.0.2 (https://primalscheme.com). The reaction mixture of 50 μL was composed of 0.5 μL of each primer (25 μM), 25 μL of 2X reaction mix (a buffer containing 0.4 mM of each dNTP, 3.2 mM MgSO_4_), 2 μL of SuperScript III RT/Platinum Taq Mix, and 14 μL of molecular grade distilled water. Subsequently, 5 μL of mumps RNA sample was added to the reaction mixture, and amplification was performed in a thermal cycler using the following program: one cycle at 50°C for 30 min, one cycle at 95°C for 10 min, and 35 cycles of 95°C for 15 s, 58°C for 30 s, and 72°C for 1 min. A final extension step at 72°C for 5 min was also included.

After amplification, samples were purified with AMPure XP magnetic beads (Beckman Coulter, Fuller-ton, USA). DNA libraries of five mumps samples were prepared following the manufacturer's instructions of the rapid barcoding kit (RBK-004, ONT). Then, the pooled samples were loaded onto a FLO-MIN106 R10.4.1 flow cell following the manufacturer's instructions (ONT). Base-calling data were retrieved from Dorado software, which is integrated into the Mk1C machine. The FASTQ files of all samples were processed and converted to FASTA files using minimap2, SAMtools, and iVar software.

### 2.4. Vaccination Cold Chain and Environmental Investigation

To identify potential sources and to further identify possible risk factors. A qualitative unstructured interview was conducted with stakeholders, including vaccine suppliers, doctors, nurses, and students. The stakeholders participated in a face-to-face group discussion focused on the vaccination cold chain and the vaccination campaign. The group included two teachers, two infection control nurses, two hospital physicians, one infectious disease physician, one pharmacist, and three vaccine suppliers. Topics discussed included a summary of the event, potential causes, the quality and standards of the MMR vaccine, the cold chain, and the vaccination campaign settings. The discussion lasted approximately 70–80 min. Additionally, in-depth interviews were conducted using purposive sampling among PNS who reported waiting more than 5 min for vaccination (due to being a significant risk in the multivariable analysis). The interviews were conducted by phone with nine PNS and two teachers after the students completed the questionnaire to gather detailed information about the vaccination campaign settings. The authors separately took notes during the interview with no audio recording. Data saturation was reached when no new information emerged, and two additional interviews were conducted to confirm saturation. Each interview lasted 5–15 min. Furthermore, a direct observation of the vaccine storage and campaign site was done. The observation focused on vaccine storage, transportation, and any possible gaps that may be a risk factor for the events.

### 2.5. Statistical Analysis

Data analysis was performed using R Version 4.4.1. Baseline characteristics were examined with descriptive statistics. Continuous data were presented as means with standard deviations (SD) or medians with interquartile ranges (IQR), where appropriate and categorical data were expressed as percentages. Differences in categorical exposure were compared using the Chi-square and Fisher's exact tests. The Wilcoxon rank-sum test was used for continuous data. Risk factors were analyzed using univariable and multivariable logistic regression, selecting variables based on background knowledge and a *p* value < 0.20 in the univariable logistic regression model. The history of previous MMR vaccination was included in the multivariable model. Nevertheless, 41 (38.0%) participants reported an “unknown” vaccination history. Therefore, three methods were applied to participants who reported an unknown history of previous MMR vaccination. These methods included (1) excluding participants who answered “unknown,” (2) using multiple imputation with a logistic regression model through the “MICE” package, with age, gender, time from retrieving the vaccine vial to vaccination, and alcohol as the predictors for the analysis which was carried out across the 40 imputed datasets, and (3) imputing with the mode of the overall responses [14]. Crude odds ratio (OR) and adjusted OR (aOR) were presented with 95% CI. A two-sided *p* value less than 0.05 was considered statistically significant.

## 3. Results

### 3.1. Confirmation of Outbreak and Diagnosis and the Outbreak Epidemiological Characteristics

To identify the baseline incidence of wild-type mumps infections, ICD-10 extraction was performed. From July 1 to August 31, 2024, two cases of salivary gland diseases (K11) were identified. Both patients presented with a sore throat but did not exhibit jaw or cheek tenderness. Hence, the patient did not meet the mumps case definition. Moreover, among the hospital service cases in the outpatient department, 61 individuals received MMR from the suspected vaccine batches at the outpatient department, of which 30 were contactable, and none met the case definition.

**Figure 1:**
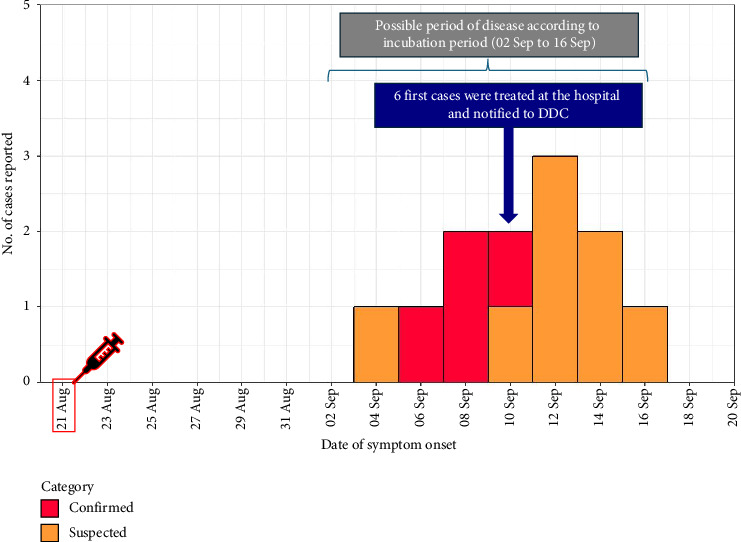
Epidemic curve of adverse event following immunization clusters of MMR vaccine among practical nursing students, Aug 21, 2024, to Oct 31, 2024 (*n* = 12); DDC: Department of Disease Control.

**Figure 2:**
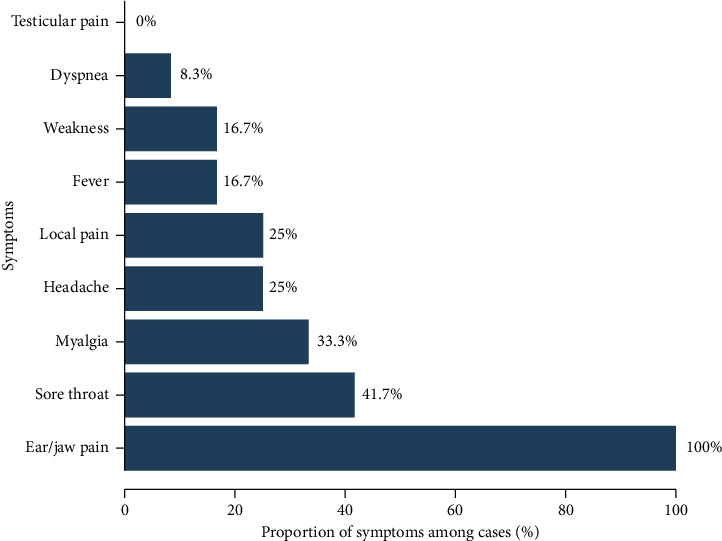
Symptoms among cases of adverse event following immunization clusters of MMR vaccine among practical nursing students, Aug 21, 2024, to Oct 31, 2024 (*n* = 12).

Active case findings were conducted in PNS and hospital service cases that received two suspected vaccine batches. Among the 108 PNS vaccinated on August 21, 2024, all responded to the questionnaire, and 12 cases were identified (eight suspected and four confirmed), resulting in an attack rate of 11.1%. Of these cases, two were male and 10 were female, with gender-specific attack rates of 12.5% for males and 10.9% for females. The first case presented with symptoms on September 4, and the last case on September 15, indicating an incubation period of 13–24 days. The epidemic curve is displayed in Figure 1. All cases reported ear or jaw pain, with 41.7% experiencing sore throats and 33.3% reporting myalgia; however, none exhibited severe symptoms (Figure 2). No severe complications were reported among the cases. One patient had an underlying condition of glucose-6-phosphate dehydrogenase (G6PD) deficiency; however, his physical examination showed no signs of jaundice or pallor. Furthermore, no mumps-like symptoms among the patients' household contacts were reported.

#### 3.1.1. Laboratory Study

**Figure 3:**
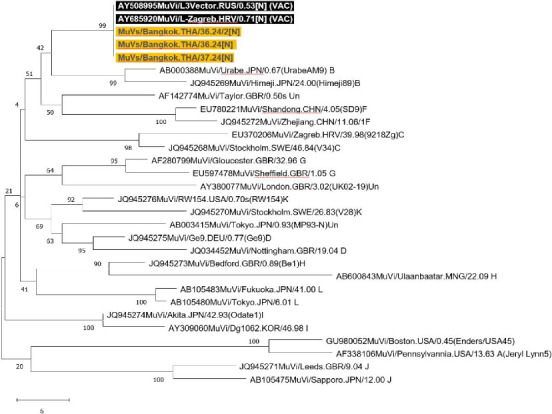
Phylogenetic tree of sequenced mumps-positive cases of partial small hydrophobic (*SH*) gene among practical nursing students. The percentage of replicate trees where the associated taxa clustered together in the bootstrap test (1000 replicates) is shown next to the branches. The tree is depicted to scale, with branch lengths in the units of the number of changes over the whole sequence. The scale bar indicates the number of nucleotides.

Twelve buccal swab specimens from all identified cases were collected, and four tested positive for mumps via RT-PCR. The median time from symptom onset to specimen collection was 2.5 days (min, max: 1–4 days) for the RT-PCR positive group and 1.5 days (range: 0–6 days) for the RT-PCR negative group. Furthermore, all RT-PCR positive cases had an earlier onset of 14–19 days. *SH* gene sequencing was performed on these four specimens, confirming that the strain belonged to the L-Zagreb strain.  Figure 3 presents a phylogenetic tree illustrating that the *SH* gene of the three RT-PCR positive cases with sufficient genetic material aligns with the L-Zagreb strain (Accession AY685920). Of the 12 cases, serum IgM for mumps was positive in four cases (33.3%), but these results were inconsistent with RT-PCR-positive cases. Serum IgG was positive in 10 cases (83.3%).  Table 1 presents the laboratory results, which align with the onset and symptoms of the patients.

#### 3.1.2. Amplicon-Based Nanopore Sequencing of Mumps NP Gene

Regarding amplicon-based nanopore sequencing of the mumps *NP* gene, no polymorphisms were detected in the collected vaccine. However, due to insufficient genetic material, identifying potential polymorphisms was not feasible.

### 3.2. Identification of Potential Sources

As there was no evidence of a wild-type mumps outbreak within the hospital or mumps-like symptoms among outpatients who received MMR, the incident is likely related to the vaccination campaign settings among PNS. Consequently, an environmental investigation of the vaccination cold chain and campaign settings was conducted to identify the potential source of the MMR-related mumps outbreak.

#### 3.2.1. Vaccination Cold Chain and Vaccine Distribution

The vaccine was manufactured by the Serum Institute of India, incorporating the L-Zagreb strain of mumps as one of its components. Two suspected batches have been identified: Lot 0133N010B (received on July 15, 2024, expiration date: August 31, 2025) and Lot 0133N053B (received on August 6, 2024, expiration date: February 28, 2026). The Thai Food and Drug Administration (FDA) has approved the vaccine since 2003, and the vaccine supplier meets the Good Distribution Practices for the Pharmaceutical Industry.

One hundred vaccine vials per lot were distributed to this hospital, totaling 200 vials. Of these, 61 doses were administered to hospital service recipients, and 108 were given to PNS on August 21, 2024. The vaccine was stored in a refrigerator, and temperature monitoring was conducted using the refrigerator's built-in thermometer sensor and an additional sensor placed inside, which alerts the pharmacist if the temperature goes out of range. This setup was aimed to maintain a temperature range of 4°C–8°C. Stock checks were performed at least daily, utilizing the first-in, first-out method to ensure that older vaccines were used first. The vaccine was transported to the vaccination site in an icebox, surrounded by ice packs on five sides but without temperature monitoring during transport.

Unlike in the vaccination campaigns, at the outpatient department, the vaccines administered to hospital service recipients were given case by case. Furthermore, the suspected vaccine batches were also distributed to four other hospitals, totaling 24,600 vials administered to adult and pediatric hospital service recipients. However, none of the recipients reported jaw or cheek pain to the hospitals.

#### 3.2.2. Vaccination Campaign Settings

**Figure 4:**
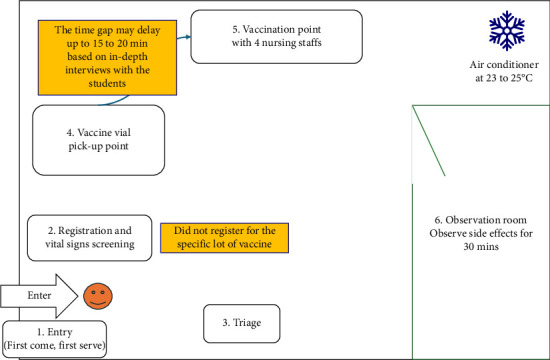
Flow of vaccination campaign and areas for improvement among practical nursing students.

According to the group discussion, the vaccination campaign was conducted twice a year using similar methods for pharmacy students and PNS. Approximately 40 pharmacy students and 100 PNS were vaccinated annually for three years, with no previous reports of AEFI clusters. However, about one to two students per year reported symptoms such as fever or cheek pain to their teacher. The vaccination campaign consisted of six stations: (1) Entry, based on first-come, first-served; (2) registration and vital sign screening; (3) triage; (4) vaccine pick-up, where students placed the vial in an aluminum tray and exposed it to room temperature while in their possession; (5) vaccination, where nursing staff mixed and administered the MMR vaccine; and (6) observation, where students were monitored for 30 min for any immediate side effects. The flow of the vaccine campaign is illustrated in Figure 4. The teachers noted that the vaccination registration did not record which vaccine lot each student received, making it difficult to determine which batch each case was vaccinated from.

### 3.3. Identification and Analysis of Possible Risk Factors

To identify potential risk factors, a retrospective cohort study was conducted among vaccinated PNS students, followed by in-depth interviews.

This study examines the relationship between demographics, preparation before vaccination, vaccination settings, previous immunization history, and MMR-related mumps infection.

Of the 12 cases, two (22.2%) were male and 10 (83.3%) were female. Eight (66.7%) reported drinking alcohol at least once a month (*p*=0.100). Regarding the time from retrieving the vaccine vial to vaccination, five cases (41.7%) reported waiting less than 5 min, two (16.7%) waited 5–15 min, and five (41.7%) waited over 15 min (*p* < 0.001). As for previous immunization history, three cases (25.0%) reported having received the MMR vaccine before and two (16.7%) reported not having received MMR before. In contrast, seven (58.3%) could not remember or did not have evidence of prior MMR vaccination (Table 2).

Multivariable logistic regression analysis adjusted for the current alcohol drinker, time to vaccination and previous history of MMR vaccine revealed that those who wait 5–15 min (aOR = 16.30, 95% CI: 1.46, 193.00) and longer than 15 min (aOR = 26.90, 95% CI: 4.20, 247.00) for vaccination are at higher risk for MMR-related mumps infection (Table 3). Those with previous immunization history of MMR may also be associated with MMR-related mumps infection (aOR = 12.90, 95% CI: 1.39, 172.00). Further imputation analysis revealed a significant association between previous MMR immunization history and the risk of MMR-related mumps infection in the multiple-imputation model (Table 4). The aOR for time to vaccination in the multivariable logistic regression with multiple imputations was slightly different from the aOR in the primary analysis but remained the same regarding the strength of association and statistical significance.

#### 3.3.1. In-Depth Interview About the Vaccination Settings

In-depth interviews were conducted with nine students (six cases and three noncases) who answered: “waited over 5 min for vaccination.” The students reported that the vaccination process involved varying wait times, depending on the queue length. One student stated, “*When the queue is long, the waiting time is approximately 15 min from receiving the vaccine vial to the actual vaccination, particularly when there are around 5 to 10 people ahead*” (ID1). Nevertheless, another noted, “*The last person in line doesn't wait as long, typically just 1 to 2 min for the provider to mix the vaccine*” (ID2).

Overcrowding was also mentioned, especially during peak times, as one student explained, “*Due to the first-come, first-served system, it sometimes gets overcrowded, especially during the middle of the vaccination period*” (ID5). Following the outbreak, a subsequent Tdap vaccination campaign was conducted. A teacher commented on the improved workflow during the campaign, “*After workflow improvements were implemented, with 20 students per round and recording time from vaccine receipt to administration, the estimated waiting time was about 6 to 7 min. The maximum wait time was 10 min, and the minimum was 3 min. Though no vaccine mixing was required during this session*.” Furthermore, regarding the temperature, the hospital's air conditioner is set to 23–25°C. Students also noted that the room felt chilly, possibly colder than 25°C.

### 3.4. Action Taken

The hospital immediately suspends the use of the suspected MMR vaccine lot and closely monitors for potential cases of MMR-related AEFI linked to these batches according to the case definitions. At the Practical Nursing School, affected students were required to undergo home isolation for 5 days after the onset of parotitis. Surveillance was established among PNS and their close contacts, with instructions to promptly report any household members exhibiting mumps-like symptoms to the teachers until October 31, 2024. Furthermore, the Department of Disease Control has informed the Thailand FDA to track the distribution of the suspected vaccine lots. Nevertheless, no other adverse events related to the L-Zagreb vaccination have been reported to the Thailand FDA in 2024. The second dose of MMR (Jeryl Lynn mumps strain) for PNS was given on October 22, 2024, under the revised operations to improve the vaccination campaign setting.

## 4. Discussion

This outbreak investigation not only confirmed the diagnosis and identified the likely source but also provided a comprehensive epidemiological assessment, analysis of risk factors, and context-specific recommendations relevant to public health practice. The clinical presentation was consistent with MMR-related mumps infection and was supported by *SH* gene sequencing of buccal swabs. The observed attack rate of 11.1% is notably higher than rates reported in regular MMR-related adverse events, highlighting the potential risk associated with certain operational aspects of mass vaccination settings [10]. Two key issues were identified: (1) the lack of individual-level vaccine lot tracking during the campaign, in contrast to standard outpatient services and (2) a delay of more than 5 minutes between vaccine vial retrieval and administration, which was significantly associated with increased infection risk. A previous history of MMR vaccination may also be a contributing factor. These findings underscore the importance of improving campaign logistics and monitoring in adult vaccination programs, particularly in tropical settings, and offer actionable insights for preventing similar adverse event clusters in the future.

Adverse events following MMR immunization may result from various mechanisms, including immune-mediated, viral/bacterial activity, injection-related, psychological/stress reactions, and coincidental events [15]. In this study, cases presented with parotitis, a symptom more specific to mumps, prompting an investigation to differentiate between MMR-related mumps infection and a wild-type mumps outbreak. However, active case findings indicated that a wild-type outbreak was unlikely. Although bacterial infection or contamination was initially considered, the clinical presentation and incubation period were consistent with mumps, and all patients exhibited mild symptoms, recovering fully without the need for antibiotics. Moreover, *SH* gene sequencing confirmed the L-Zagreb vaccine strain in multiple cases, supporting a viral etiology. Additionally, the information from the interviews suggested that the handling process during vaccination was appropriate. Hence, bacterial contamination was less likely to occur. While several MMR vaccination campaigns have resulted in similar side effects, only one previous study has confirmed a vaccine-related mumps infection through molecular diagnostic techniques [3, 11].

The MMR vaccine is a live-attenuated vaccine that, despite its weakened state, carries a potential risk of viral replication and infection, particularly in individuals with immunodeficiency [16]. Among its components, the mumps virus presents the most significant challenge in achieving optimal attenuation while ensuring long-term immunity, making it the most difficult to attenuate effectively [17, 18]. Thus, suboptimal attenuation may occur, increasing the risk of MMR-related mumps infection if the vaccine is not consistently stored under standard conditions [11].

The attack rate in this study was 11.1%, which is comparable to rates reported in previous vaccination campaigns in Thailand (9.3%) and Suriname (15.1%) using the L-Zagreb strain. The Thai campaign targeted adults aged 20–21 years, while the Surinamese campaign focused on children around 10 years [3, 11]. These attack rates are generally higher than those previously reported for the MMR vaccine [10]. Another MMR mass immunization campaign using the L-Zagreb strain, conducted in January 2009 on 1875 young adults at a military recruitment center in Turkey, resulted in 10 cases of parotitis [8]. Despite using the same vaccine strain and targeting a similar adult population, attack rates varied across events. Factors such as vaccination procedures, storage, transportation, and meteorological conditions—which were unreported in previous studies—may have contributed to these fluctuations. Furthermore, to our knowledge, no other mumps strain in the MMR vaccine has been linked to MMR-related mumps infection in immunization campaign settings to date.

The analysis in the present study suggests a possible association between delayed vaccine administration, specifically a delay of more than 5–15 min after retrieving the vaccine vial at room temperature (23°C–25°C), and an increased risk of adverse events. While the mumps component in MMR vaccines has shown to remain potent at 37°C for up to 21 days, the impact of ambient temperature on viral replication dynamics in field conditions has not been thoroughly studied [19]. The recommended storage temperature for MMR vaccines is 2°C–8°C, and deviations from this range may potentially affect viral behavior [20].

Environmental studies have shown that higher temperatures may enhance mumps virus activity, with one study from Guangzhou reporting increased activity with temperature rises, and another from Taiwan demonstrating an uptick in mumps cases at 20°C that declines above 25°C [20, 21]. The relatively higher rates of parotitis reported in Thailand and Suriname, compared to cooler regions such as Turkey, may be partially explained by tropical environmental conditions, which could contribute to temperature-related changes in vaccine virus activity during mass campaigns [3, 8, 11]. Notably, no parotitis cases were observed among hospital outpatients who received vaccines from the same lots but under routine cold-chain–controlled settings. Although a causal mechanism remains speculative, these findings warrant further investigation into the role of temperature fluctuations and administration delays in the context of live attenuated vaccines.

Typically, MMR-induced antibodies are expected to persist in the body, providing lifelong protection against mumps infection [22]. However, our findings indicated that a history of MMR vaccination was associated with MMR-related mumps infection, contrasting with the expected immune response. This discrepancy could be attributed to potential genetic polymorphisms in the mumps virus that may occur after MMR vaccination, potentially increasing viral fusogenicity and reducing the effectiveness of pre-existing immunity [3, 23]. Nevertheless, further studies are needed before considering any changes to MMR vaccination recommendations for individuals with prior MMR vaccination. Additionally, our findings regarding the potential risk associated with previous MMR vaccination are not yet conclusive, as the statistical significance varied across different methods of handling missing data.

Ensuring immunity among healthcare workers is still critical, and prevaccination serological testing may present a cost-effective strategy. A study conducted in the United States reported a comparison between the cost of serological testing for mumps, measles, and rubella ($57.99) and the expense of administering two doses of the MMR vaccine ($104.14) [24, 25]. However, costs may vary by country; while serology can indicate immunity per vaccination guidelines, it does not always correlate with protective immunity [25].

The present study found that only four out of 12 cases tested positive for mumps via RT-PCR. This may be due to the difficulty in obtaining sufficient clinical genetic material for mumps, as noted in previous studies [3]. RT-PCR for mumps yields the highest rate of positive results, ranging from 70% to 80% when conducted within 3 days of parotitis onset. However, this rate decreases to approximately 50% if testing occurs after 3 days, as observed in wild-type mumps outbreaks [26]. Moreover, evidence suggests that virus levels in vaccinated individuals with mumps infection may be low and only briefly detectable, even in cases presenting with parotitis. This may lead to increased false-negative results, even when testing is conducted within 3 days, as observed in the present study [27–29]. In addition, all RT-PCR-positive cases were from the early onset group, suggesting a potentially higher positivity rate among individuals with earlier onset. Hence, swabbing the parotid duct and buccal cavity after a 30-s parotid massage or with multiple buccal swabs per specimen may optimize viral detection and yield [26, 30]. Regarding IgM and IgG testing, using serology tests to differentiate between seroconversion due to vaccination, MMR-related infection, or wild-type mumps infection is particularly challenging in a vaccination campaign setting [28]. Therefore, routine serological testing for mumps may not be necessary in the present context.

### 4.1. Recommendations

Our study provides several key insights and recommendations. First, improving vaccine registration during mass vaccination campaigns is essential for accurately tracking AEFI outbreaks, such as incorporating the vaccine lot number into the registration process. Second, strategies should be implemented to minimize the time between retrieving the vaccine vial and administering the vaccination. For example, keeping the vial with the vaccine administrator could streamline the process. Third, alternative vaccine options with lower adverse events, such as different MMR strains, might also be considered for future immunization campaigns, particularly among adults [10]. Fourth, serological testing for mumps may not be recommended in vaccine-related mumps infection outbreaks. Fifth, the lower viral yield in MMR-related mumps infections compared to wild-type infections suggests that swabbing the parotid duct and buccal cavity after a 30 s parotid massage or using multiple buccal swabs per specimen may enhance viral detection. Last, future research should focus on identifying potential polymorphisms in the L-Zagreb strain that may contribute to increased virulence, possibly through functional studies. Further studies should investigate the effects of temperature variations on the virulence of the L-Zagreb strain, primarily through in vitro experiments. Moreover, although long-term complications have not been observed in this outbreak, studies should explore whether MMR-related mumps infections, such as wild-type infections, carry any risk of long-term sequelae such as sensorineural hearing loss [31].

### 4.2. Strengths and Limitations

This study offers important public health insights from a confirmed cluster of MMR-related mumps infection, supported by *SH* gene sequencing that identified the L-Zagreb vaccine strain as the source. It integrates epidemiological, molecular, and operational methods, including active case finding, retrospective cohort analysis, and multiple imputations to strengthen the validity of findings. A key strength is the identification of a modifiable operational factor, delayed vaccine administration, which may help improve campaign workflows. The study also addresses challenges in vaccine lot tracking and AEFI surveillance among adults, a population often overlooked in vaccine safety research. These findings support practical recommendations and highlight the need for future research on administration timing, temperature exposure, and repeated MMR vaccination.

Nevertheless, this study has several limitations that should be acknowledged. First, recall and memory biases from the students could have influenced the accuracy of the reported information. The semistructured questionnaire relied on subjective responses, potentially introducing social desirability bias, as participants may have provided answers they believed were more acceptable. Additionally, although participants were asked to provide their vaccination books to document their previous vaccination history, only 27 out of 67 participants (40.3%) had their history recorded using childhood vaccination records. Furthermore, while an association between prior MMR vaccination and outcomes was observed, the validity of this finding is limited by the high proportion of participants (38.0%) who were unable to recall their vaccination history. However, multiple imputation analyses were conducted to address this limitation. The positive association also remains the same in all models after imputation. Second, the joint investigation team could not directly observe the mass vaccination process, which may have limited our ability to identify procedural issues in real time. Although contamination or bacterial infection was considered, the clinical presentation was consistent with mumps, and molecular testing confirmed the L-Zagreb vaccine strain in multiple cases, supporting a viral etiology. Third, MMR-related mumps infection among hospital service recipients may be underestimated, as mild symptoms might not have been reported. Nonetheless, we also conducted active case finding, which revealed that none of the 30 contactable vaccinated patients met the case definition. Finally, the patient's genetic material was insufficient for whole-genome sequencing (WGS), limiting further analysis of potential viral mutations. Previous studies have shown that WGS can enhance outbreak investigations and identify essential differences between vaccine and circulating strains [32]. This highlights the importance of meticulous specimen collection in cases of MMR-related mumps infection to ensure the highest possible yield for genetic analysis. However, given the urgency of outbreak investigation and the resources available, *SH* gene sequencing is a more feasible option, albeit with a slightly compromised resolution compared to WGS.

## 5. Conclusion

This study confirmed that the outbreak was caused by MMR-related mumps infection. The observed attack rate was comparable to previous vaccination campaigns using the L-Zagreb strain MMR vaccine among adults in tropical countries. However, it was relatively higher than rates reported in other settings. A prolonged interval between retrieving and administering the vaccine may have led to temperature changes, potentially increasing the rate of MMR-related mumps infections; however, further studies are required to substantiate this hypothesis. Based on these findings and existing evidence, the use of the L-Zagreb strain MMR vaccine should be approached with caution in adults, especially in mass vaccination campaign settings.

## Figures and Tables

**Table 1:** Clinical presentation and laboratory tests of the MMR-related adverse events cases (*n* = 12).

Patient	Onset	Specimen collection date	Age	Presenting symptoms	Serum IgM for mumps	Serum IgG for mumps	RT-PCR for mumps	SH-protein sequencing
1	4/9/2024	10/9/2024	20	Tender at right cheek and ear	**Positive**	**Positive**	Negative	Not done
2	6/9/2024	10/9/2024	19	Tender at left cheek and ear, fever	Negative	**Positive**	**Positive**	Too little DNA
3	7/9/2024	10/9/2024	20	Tender at right cheek and ear	Negative	**Positive**	**Positive**	**L-Zagreb**
4	8/9/2024	10/9/2024	25	Tender at right cheek	**Positive**	**Positive**	**Positive**	**L-Zagreb**
5	9/9/2024	10/9/2024	19	Tender at left cheek, fever, and sore throat	Negative	Negative	Negative	Not done
6	9/9/2024	10/9/2024	18	Tender at left cheek and fever	**Positive**	**Positive**	**Positive**	**L-Zagreb**
7	11/9/2024	11/9/2024	18	Tender at left cheek	Negative	**Positive**	Negative	Not done
8	12/9/2024	13/9/2024	20	Sore throat, tender at left ear	Negative	**Positive**	Negative	Not done
9	12/9/2024	13/9/2024	19	Tender left jaw, sore throat	Negative	**Positive**	Negative	Not done
10	13/9/2024	16/9/2024	18	Tender left jaw, sore throat	Negative	**Positive**	Negative	Not done
11	14/9/2024	17/9/2024	20	Tender left cheek, fever, sore throat, cough	**Positive**	Borderline	Negative	Not done
12	15/9/2024	17/9/2024	24	Mild tender right jaw, fever, sore throat, cough	Negative	**Positive**	Negative	Not done

*Note:* Bold indicates a positive laboratory result.

**Table 2:** Characteristics of practical nursing students stratified by MMR-related mumps infection case (*N* = 108).

Characteristics	^a^Noncase	^a^Case	^b^ *p* value
*Demographics*			
Gender			> 0.999
Female	82 (89.2%)	10 (10.8%)	
Male	14 (87.5%)	2 (12.5%)	
Age (median (Q1, Q3))	19.00 (18.0, 22.0)	19.50 (18.8, 20.0)	0.852
BMI (median (Q1, Q3))	21.1 (18.5, 23.9)	22.0 (19.8, 23.7)	0.694
Current alcohol drinker (at least once a month)	40 (83.3%)	8 (16.7%)	0.100
Current smoker	7 (100.0%)	0 (0.0%)	> 0.999

*Preparation before vaccination (48 h prior)*			
Sleep less than 7 h	36 (87.8%)	5 (12.2%)	0.763
Vigorous exercise	8 (100.0%)	0 (0.0%)	> 0.999
Drink alcohol	3 (100.0%)	0 (0.0%)	> 0.999

*Vaccination stage*			
Time period receiving the MMR vaccine			0.015
1300–1400	22 (91.7%)	2 (8.3%)	
1400–1500	37 (94.9%)	2 (5.1%)	
1500–1600	16 (100.0%)	0 (0.0%)	
Can't remember	24 (80.0%)	6 (20.0%)	
Time from retrieving the vaccine vial to vaccination			< 0.001
< 5 min	89 (94.7%)	5 (5.3%)	
5–15 min	3 (60.0%)	2 (40.0%)	
> 15 min	4 (44.4%)	5 (55.6%)	

*Immunization history*			
Previous history of MMR vaccine			0.063
No	48 (96.0%)	2 (4.0%)	
Yes	13 (81.3%)	3 (16.7%)	
Unknown	35 (83.3%)	7 (16.7%)	
Positive for varicella immune	49 (90.7%)	5 (9.3%)	0.852
Positive for hepatitis B immune	19 (86.4%)	3 (13.6%)	0.694

^a^ *n* (%); median (IQR).

^b^Pearson's Chi-squared test or Fisher exact test for categorical variable and Wilcoxon rank sum test for continuous variables.

**Table 3:** Univariable and multivariable logistic regression analysis of possible risk factors toward MMR-related mumps infection.

Characteristics	OR	95% CI	*p* value	aOR^∗^	95% CI	*p* value
Current alcohol drinker						
No	Ref	Ref		Ref	Ref	
Yes	2.80	0.82, 11.10	0.110	2.39	0.48, 13.60	0.300
Time to vaccination						
< 5 min	Ref	Ref		Ref	Ref	
5–15 min	**11.90**	**1.34, 90.20**	**0.016**	**16.30**	**1.46, 193.00**	**0.020**
> 15 min	**22.20**	**4.64, 120.00**	**< 0.001**	**26.90**	**4.20, 247.00**	**0.001**
Previous history of MMR vaccine						
No	Ref	Ref		Ref	Ref	
Yes	5.54	0.84, 45.50	0.076	**12.90**	**1.39, 172.00**	**0.030**
Unknown	4.80	1.08, 33.50	0.059	4.53	0.74, 45.20	0.130

*Note:* Bold values indicate statistical significance of *p* < 0.05.

Abbreviations: aOR = adjusted odds ratio, CI = confidence interval, and OR = odds ratio.

^∗^Adjusted for the current alcohol drinker, time to vaccination, and previous history of MMR vaccine.

**Table 4:** Multivariable logistic regression analysis of possible risk factors toward MMR-related mumps infection with imputation of previous history of MMR vaccine.

Characteristics	Remove unknown			Multiple imputation^∗∗^			Impute unknown with mode		
	aOR^∗^	95% CI	*p* value	aOR^∗^	95% CI	*p* value	aOR^∗^	95% CI	*p* value
Current alcohol drinker									
No	**Ref**			**Ref**			**Ref**		
Yes	**6.83**	0.96, 63.60	0.059	2.86	0.55, 15.00	0.545	2.94	0.63, 16.30	0.200
Time to vaccination									
< 5 min	**Ref**	Ref		Ref	Ref		Ref	Ref	
5–15 min	**N/A**	N/A	N/A	**21.28**	**2.72, 166.35**	**0.004**	**26.20**	**2.46, 296.00**	**0.005**
> 15 min	**3.43**	**0.12, 49.00**	**0.400**	**21.78**	**1.41, 336.14**	**0.029**	**23.10**	**4.11, 158.00**	**< 0.001**
Previous history of MMR vaccine									
No	**Ref**	Ref		Ref	Ref		Ref	Ref	
Yes	6.83	0.96, 63.60	0.059	**7.63**	**1.02, 57.03**	**0.048**	5.57	0.82, 37.90	0.069

*Note:* Bold values indicate statistical significance of *p* < 0.05.

Abbreviations: aOR = adjusted odds ratio and CI = confidence interval.

^∗^Adjusted for the current alcohol drinker, time to vaccination, and previous history of MMR vaccine.

^∗∗^Multiple imputations using the logistic regression method by the “MICE” package in R.

## Data Availability

The datasets used and/or analyzed in this study are available from the authors on reasonable request (via Sethapong.ler@pcm.ac.th).

## Nomenclature

PNS: Practical nursing student
RT-PCR: Reverse transcription polymerase chain reaction
MMR vaccine: Measles–Mumps–Rubella vaccine
SH: Small hydrophobic
NP: Nucleoprotein
AEFI: Adverse event following immunization
L-Zagreb: Leningrad-Zagreb
